# A Comprehensive Approach to Polycystic Ovarian Syndrome: A Case Report of Successful Intracytoplasmic Sperm Injection Treatment Utilizing the Short Antagonist Protocol and Hatching

**DOI:** 10.7759/cureus.54457

**Published:** 2024-02-19

**Authors:** Nancy Nair, Akash More, Nandkishor J Bankar, Ankit Badge, Ujwal Gajbe, Brij Raj Singh

**Affiliations:** 1 Clinical Embryology, School of Allied Health Sciences, Datta Meghe Institute of Higher Education and Research, Wardha, IND; 2 Clinical Embryology, Wardha Test Tube Baby Centre, Datta Meghe Institute of Higher Education and Research, Wardha, IND; 3 Microbiology, Jawaharlal Nehru Medical College, Datta Meghe Institute of Higher Education and Research, Wardha, IND; 4 Microbiology, Datta Meghe Medical College, Datta Meghe Institute of Higher Education and Research, Nagpur, IND; 5 Anatomy, Datta Meghe Medical College, Datta Meghe Institute of Higher Education and Research, Nagpur, IND

**Keywords:** gnrh antagonist protocol, intra-cytoplasmic sperm injection, mechanical hatching, anovulation, polycystic ovarian syndrome

## Abstract

The 29-year-old participant in the case study has been grappling with infertility for the last six years. Following an assessment of her symptoms, hormone profile, and ultrasound results, she received a diagnosis of polycystic ovarian syndrome (PCOS). PCOS is a multifaceted endocrine and metabolic disorder characterized by symptoms such as obesity, insulin resistance, anovulation, and polycystic ovaries. Various factors, including heredity, intestinal dysbiosis, obesity, environmental pollutants, lifestyle choices, and neuroendocrine abnormalities, contribute to the susceptibility of women to PCOS. In planning polycystic ovarian stimulation, it is crucial to consider parameters such as antral follicle count (AFC), luteinizing hormone (LH), and anti-Müllerian hormone (AMH). Careful planning of the gonadotrophin dose is essential to achieve an optimal response during a gonadotropin-releasing hormone antagonist (GnRH-ant) cycle. In our case, the brief antagonist protocol was used, resulting in a favorable outcome with minimal risk of ovarian hyperstimulation syndrome (OHSS). Despite multiple unsuccessful attempts at natural conception, the patient successfully conceived with the help of intracytoplasmic sperm injection (ICSI), leading to a positive pregnancy outcome. In addition to incorporating mechanical hatching to promote implantation, we diligently selected the most beneficial medications for the patient.

## Introduction

A couple is labeled infertile when they are unable to conceive after a year or more of unprotected intercourse. The challenge of infertility is a distressing experience for many couples, affecting various aspects of their lives, including physical, emotional, and sexual well-being [1,2]. Asherman syndrome, endometriosis, fallopian tube obstruction, polycystic ovarian syndrome (PCOS), thyroid imbalance, genital tract infections, and congenital uterine diseases are a few of the conditions that can cause infertility in women [3,4].

PCOS is estimated to affect around 4-10% of women globally within the reproductive age range. This approximation is derived from a thorough screening process that adheres to the diagnostic criteria established by the National Institutes of Health (NIH) [5]. Oligomenorrhea, sometimes accompanied by amenorrhea, is a characteristic hallmark of this syndrome. PCOS is associated with infertility and insulin intolerance. Additionally, it causes various irregularities in the female body and abnormalities in the reproductive tract. PCOS stands out as one of the prevalent anovulatory factors that contribute to infertility [5,6].

Embryo transfer (ET) and in vitro fertilization (IVF) are the most widely adopted infertility treatments worldwide. The intricate IVF process involves various stages, beginning with gonadotropin-stimulated ovarian stimulation, followed by oocyte harvesting, laboratory fertilization, embryo culture, and culminating in ET to the uterus. To enhance the chances of a successful pregnancy, the first crucial step is to initiate the maturation of multiple oocytes through controlled ovarian stimulation (COS). However, the challenge lies in managing multifollicular development, which often triggers an unexpected increase in luteinizing hormone (LH) and an early increase in estradiol levels. This phenomenon affects a considerable percentage (12.3-46.7%) of new IVF cycles, and emerging evidence suggests its detrimental impact on overall success rates. For individuals struggling with infertility caused by PCOS-related anovulation, the preferred initial pharmacological intervention commonly involves inducing ovulation. This can be achieved by using the aromatase inhibitor letrozole or oral anti-estrogen clomiphene citrate [7]. IVF treatment is recommended as an effective final option if ovulation induction, including the use of second-line gonadotropin medications, proves ineffective in achieving a viable pregnancy or if the patient experiences infertility due to tubal and/or male factors. For individuals with PCOS, gonadotropin-releasing hormone antagonist (GnRH-ant) protocols are considered a safer alternative to standard GnRH agonist protocols. This is because they decrease the risk of ovarian hyperstimulation syndrome (OHSS), to which PCOS patients are particularly susceptible. The use of GnRH-ant treatment offers several advantages, including a significant reduction in the occurrence of OHSS, a lower dose of medication, a shorter duration of treatment, and the absence of a "flare-up" effect [7-10].

## Case presentation

We are detailing the case of a 29-year-old woman and a 33-year-old man, both of whom have not experienced pregnancy previously and have encountered challenges in conceiving throughout the six years of their married life. The couple experienced significant emotional distress due to social pressure and criticism. Before opting for IVF, they sought the help of various healthcare facilities to address their concerns about infertility.

Medical history

The female patient has a healthy lifestyle without the habit of smoking, drinking alcohol, or any kind of exposure to drugs. Additionally, her blood glucose levels indicated the absence of diabetes, and her blood pressure fell within the normal range, indicating a normotensive state. Additionally, the complete blood count report did not show abnormalities. Both the semen tests and the examination of the patient's uterine tube confirmed no pathology. PCOS was diagnosed based on ultrasound examinations that revealed distinctive ovarian morphology, prolonged monthly cycles, and lack of ovulation. The patient's husband had received the same diagnosis from another infertility clinic earlier. A complete family history revealed the presence of PCOS on the mother's side. To address the irregular menstrual cycle, an endocrinologist prescribed hormone-balancing medications, which successfully reduced the cycle gap to 26-28 days. Despite receiving repeated doses of citrate clomiphene to stimulate ovulation, the patient was unable to conceive after three unsuccessful intrauterine insemination (IUI) treatments. For additional infertility treatment options, the patient opted for counseling.

Diagnostic assessment

The individual sought assistance at the Wardha Test Tube Baby Center, Sawangi (Meghe), in 2022, expressing concerns about infertility. Upon admission to our medical facility, the patient was advised to undergo blood examinations on the third day of her menstrual cycle to evaluate a complete hormonal profile. After admission, tests were conducted to determine the serum concentration of various hormones, including testosterone, prolactin, LH, etc., as additional diagnostic measures. Taking into account the patient's medical history and ultrasound findings, a comprehensive assessment of her hormonal profile confirmed the initial diagnosis of PCOS. Consequently, counseling was provided to the patient about IVF. Table 1 presents the hormonal profile of the women and the corresponding laboratory values, along with the reference ranges. LH is measured at 17.0 mIU/mL, exceeding the reference range of 2-15 mIU/mL. The testosterone level is 1.2 ng/mL, within the normal range of 0.15-0.7 ng/mL. Progesterone is recorded at 0.20 ng/mL, below the reference range of 1-5 ng/mL. Estradiol is 30.22 pg/mL, within the reference range of 30-400 pg/mL. The follicle-stimulating hormone (FSH) is 4.02 mIU/mL, within the normal range of 3.5-12.5 mIU/mL. Anti-Müllerian hormone (AMH) is elevated at 6.20 ng/mL, exceeding the normal range of 1.0-4.0 ng/mL. Prolactin is 12.0 ng/mL, within the reference range of 2-29 ng/mL.

**Table 1:** The hormonal profile of the female patient AMH, anti-Müllerian hormone; FSH, follicle-stimulating hormone; LH, luteinizing hormone; mIU/mL, milli-international units per milliliter; ng/mL, nanograms per milliliter; pg/mL, picograms per milliliter

Parameters	Lab value	Reference range
LH	17.0 mIU/mL	2-15 mIU/mL
Testosterone	1.2 ng/mL	0.15-0.7 ng/mL
Progesterone	0.20 ng/mL	1-5 ng/mL
Estradiol	30.22 pg/mL	30-400 pg/mL
FSH	4.02 mIU/mL	3.5-12.5 mIU/mL
AMH	6.20 ng/mL	1.0-4.0 ng/mL
Prolactin	12.0 ng/mL	2-29 ng/mL

**Figure 1:**
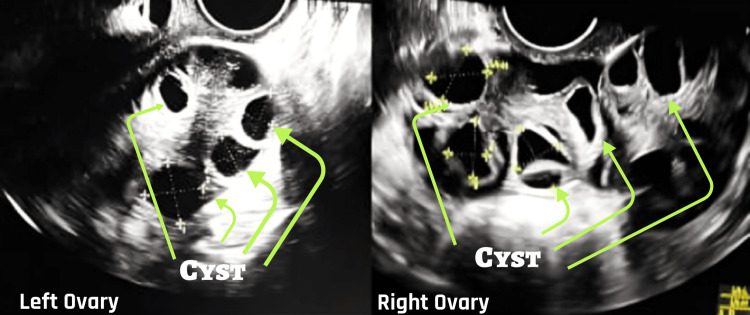
Transvaginal sonography image of patient's right and left ovary on day 2 of menstrual cycle Depict multiple cysts present in the right and left ovary.

Figure 1 shows the transvaginal sonography image of the patient's right and left ovary on day 2 of the menstrual cycle.

Treatment plan

Aggressive reactions and OHSS are more prevalent in women with PCOS and polycystic ovarian morphology (PCOM). An approach to mitigating an early LH surge is the use of GnRH agonists or GnRH-ant. Antagonists have been associated with fewer follicles, reduced estradiol levels, and a shorter stimulation period. A comprehensive meta-analysis of randomized controlled trials (RCTs) indicates that there are no significant differences in sustained pregnancy rates or live birth rates between GnRH agonists and antagonists. In particular, the incidence of severe OHSS was markedly lower in GnRH-ant cycles. Scientific studies are increasingly supporting the use of GnRH-ant in IVF for PCOS patients. Following informed consent from the patient, we initiated GnRH short-agonist treatment to minimize the risk of OHSS.

During the ovum pick-up (OPU) procedure, strict adherence to the short GnRH-ant regimen was maintained. Human menopausal gonadotropin (hMG) at a dose of 225 IU was administered on the second or fourth day of the menstrual cycle. Treatment with 0.25 mg of GnRH ganirelix antagonist commenced on the fifth day of stimulation. The hMG and GnRH-ant drugs persisted until the oocyte maturation trigger. Oocyte growth and maturity were observed every other day of stimulation. Human chorionic gonadotropin (hCG) at a dose of 5,000 IU was administered 36.5 hours before OPU to facilitate egg maturation. On the day of OPU, a total of two metaphase I (MI) and four metaphase II (MII) oocytes were recovered. Three MII oocytes fertilized, and one 3BA-quality day 5 blastocyst stage embryo was formed and vitrified. The couple was informed about the number and quality of the embryos. In preparation for transfer, the patient received oral estradiol 2 mg daily, metformin 250 mg, and cabergoline 5 mg twice a day. L-arginine sachets were also recommended. Progesterone was initiated six days before the scheduled ET, with an endometrial thickness measuring 9 mm on the transfer day.

Due to the patient's history of infertility and unsuccessful IUIs, both embryos frozen on the fifth day of intracytoplasmic sperm injection (ICSI) were thawed and mechanically hatched to potentially improve implantation. A follow-up was scheduled after ET, with instructions for the patient to avoid heavy or strenuous activities for the next 14 days.

**Figure 2:**
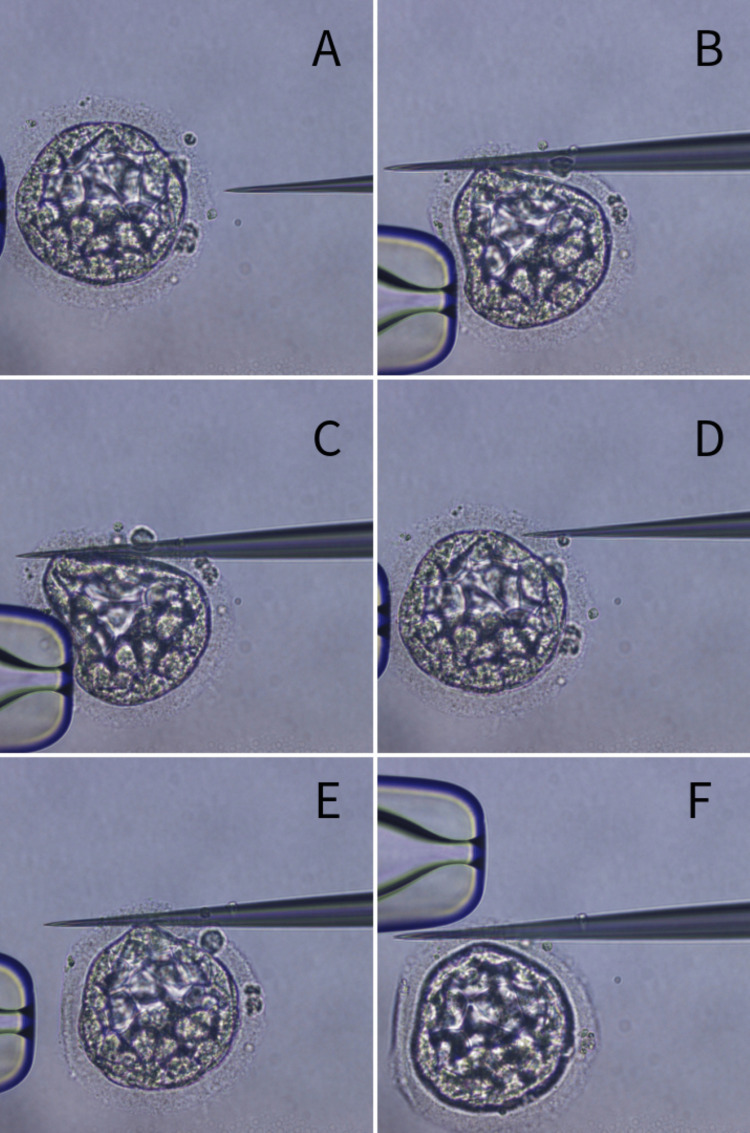
Process of mechanical embryo hatching Where the black arrow depicts the selected embryo, the red arrow depicts the hatching needle, and the yellow arrow depicts the holding. (A) Holding the selected embryo for hatching. (B) Inserting the hatching needle. (C) Hatching by needle. (D) Removal of the needle. (E) Reinserting of the needle. (F) Completing mechanical hatching.

Figure 2 shows a step-wise procedure carried out for mechanical embryo hatching of the selected embryo for transfer.

Follow-up

After ET, the patient received calcium supplements, iron, vitamins, and coenzyme Q10. A follow-up appointment was scheduled on day 14, during which the patient was advised to undergo a urine pregnancy test. The test showed a positive result, supported by a serum β-hCG reading of 200 mIU/mL. Subsequently, the patient was instructed to arrange an ultrasound examination two weeks later, confirming the presence of a single healthy fetus with a normal growth rate. Eventually, at 36 weeks of gestation, a healthy baby girl of 2.8 kg was born with a cesarean section.

## Discussion

The prevalent disease known as PCOS presents particular difficulties and limitations for the field of assisted reproductive technologies. For infertile couples, this is the most typical cause of anovulation [11]. Therefore, it is imperative to document research projects and their positive outcomes to supplement the existing data and establish a treatment strategy for patients with PCOS. According to certain studies, gonadotropins are associated with a higher risk of OHSS and multiple pregnancies in PCOS patients [8,12]. Hence, they should only be given by medical experts who possess the required training and experience. The incidence of OHSS, clinical pregnancies, multiple pregnancies, live births, and miscarriages does not exhibit any significant variation across gonadotropin preparations, FSH, or hMG [11].

Studies dispute the claims made about the superior and more clinical pregnancy outcomes associated with early GnRH agonist treatments. GnRH-ant use is becoming more and more supported in women with PCOS who are undergoing IVF. Previous worries about lower pregnancy rates with antagonists compared to agonists have been addressed as evidence is growing that they reduce OHSS rates and do not differ in pregnancy rates from agonists. Many facilities have completely shifted to treating patients with GnRH-ant [8]. This case study supports the author's findings by showing how flexible the short GnRH-ant treatment is and how well it worked for the patient. In oocyte donors who were at threat of OHSS, a prospective randomized, double-blind study examined the effects of daily cabergoline treatment (0.5 mg/day). The results showed that using cabergoline starting on the day of hCG and continuing for six days after oocyte retrieval reduced the incidence of moderate OHSS by more than 50%. Once more, this is a novel solution that has been advised in situations when there is a high threat of OHSS [13]. In agreement with this article, our patient responded positively to the medication, and there was no threat faced by the patient for OHSS. A successful pregnancy result occurred, and the risk of OHSS was avoided. Consequently, a short antagonist treatment is recommended for females with PCOS, as this case report supports. There is a substantial correlation between the probability of multiple pregnancies and clinical pregnancy among women who underwent assisted hatching [14]. Mechanical hatching was chosen in this study, which enabled the hatched embryo to implant successfully. This, in turn, adds to the literature suggesting that assisted hatching may lead to a positive pregnancy and live birth outcome.

## Conclusions

This case report presents the positive pregnancy of a PCOS patient with the administration of personalized treatment. It has been demonstrated that careful monitoring and the use of individualized treatment plans can avoid OHSS in PCOS patients. With the use of the short-agonist protocol, ovarian stimulation was controlled without any side effects on the patient. The successful use of mechanical hatching is highlighted in this case report, demonstrating its efficacy in producing positive implantation outcomes and increasing the overall success of assisted reproductive technology (ART). Even though the medications and stimulation protocol used alongside mechanical hatching worked well in this instance, additional research is needed to draw more definitive conclusions about treatment options for PCOS patients.
